# Taxonomic study of the genus *Halolaguna* Gozmány (Lepidoptera, Lecithoceridae) from China, with descriptions of two new species

**DOI:** 10.3897/zookeys.464.8571

**Published:** 2014-12-16

**Authors:** Kaijian Teng, Shurong Liu, Shuxia Wang

**Affiliations:** 1 College of Life Sciences, Nankai University, Tianjin 300071, P. R. China

**Keywords:** Lepidoptera, Lecithoceridae, *Halolaguna*, new species

## Abstract

The genus *Halolaguna* Gozmány, 1978 is studied in China. Two new species, *Halolaguna flabellata* **sp. n.** from Guangxi and *Halolaguna discoidea* **sp. n.** from Chongqing, Guangxi and Sichuan are described. The female of *Halolaguna guizhouensis* Wu, 2012 is reported for the first time. Photographs of adults and genitalia are provided. A checklist of all known *Halolaguna* species is included, along with a key to the Chinese species.

## Introduction

The family Lecithoceridae occurs particularly in the Oriental and Australian Regions, with around 1,200 described species (van Nieukerken et al. 2011). Wu (1997) recorded 206 species of Lecithoceridae from China and Park et al. (2013) listed 74 species of Lecithoceridae from Chinese Taiwan. To date, approximately 290 species of this family have been reported from China.

*Halolaguna* Gozmány, 1978 is a small genus of the subfamily Torodorinae in Lecithoceridae, which was established by Gozmány in 1978 based on the type species *Halolaguna sublaxata* Gozmány, 1978 from China. Subsequently, Wu (2000) transferred *Lecithocera biferrinella* Walker, 1864 to *Halolaguna*, and described *Halolaguna orthogonia* Wu, 2000 from Malaysia; Park (2000) transferred *Cynicostola oncopteryx* Wu, 1994 to *Halolaguna*, and described *Halolaguna palinensis* Park, 2000 from Taiwan; Park (2011) further described *Halolaguna sanmaru* Park, 2011 from Thailand; and Wu (2012) described *Halolaguna guizhouensis* Wu, 2012 from Guizhou. To date, *Halolaguna* includes seven species confined to the Oriental and Palaearctic regions, but little is known about the biology of this genus so far.

*Halolaguna* is characterized by having an elongate and relatively narrow forewing with M_2_ and M_3_ coincident, and the valva tapering to the apex in the male genitalia. *Halolaguna* is similar to *Antiochtha* Meyrick, 1905 in both appearance and male genitalia, but can be distinguished by the presence of M_2_ in the hindwing, which is absent in *Antiochtha*. It is also similar to *Athymoris* Meyrick, 1935 in the venation, but differs in the valva in the male genitalia that is tapering to a pointed apex, whereas the valva is foot-shaped and widened terminally in *Athymoris*.

We report five *Halolaguna* species from mainland China in this paper, based on the specimens collected mostly from mountainous regions and natural reserves. Two species are described as new, and the female of *Halolaguna guizhouensis* Wu, 2012 is described for the first time.

## Material and methods

The specimens examined in this study were collected from mountains, botanical gardens and nature reserves in China by light traps. All specimens studied, including the types, are deposited in the Insect Collection, College of Life Sciences, Nankai University, Tianjin, China.

Genitalia dissections were carried out following Li (2002). Photographs of the adults were taken with a Leica stereo microscope M205A plus Leica Application Suite 4.2 software, and genitalia were photographed using a Leica DM750 microscope plus the same software as for adults.

## Taxonomic accounts

### *Halolaguna* Gozmány, 1978

*Halolaguna* Gozmány, 1978: 238. Type species: *Halolaguna sublaxata* Gozmány, 1978. Type locality: China (Jiangsu).

### Checklist of *Halolaguna* species

*Halolaguna biferrinella* (Walker, 1864)

*Lecithocera biferrinella* Walker, 1864: 642.

*Halolaguna biferrinella*: Wu, 2000: 428.

Distribution. Malaysia, Indonesia.

*Halolaguna discoidea* sp. n.

Distribution. China (Chongqing, Guangxi, Sichuan).

*Halolaguna flabellata* sp. n.

Distribution. China (Guangxi).

*Halolaguna guizhouensis* Wu, 2012

*Halolaguna guizhouensis* Wu, 2012: 394.

Distribution. China (Chongqing, Guangdong, Guangxi, Guizhou).

*Halolaguna oncopteryx* (Wu, 1994)

*Cynicostola oncopteryx* Wu, 1994: 125.

*Halolaguna oncopteryx*: Park 2000: 240.

Distribution. China (Chongqing, Fujian, Guangxi, Sichuan, Taiwan, Yunnan, Zhejiang).

*Halolaguna orthogonia* Wu, 2000

*Halolaguna orthogonia* Wu, 2000: 427.

Distribution. Malaysia.

*Halolaguna palinensis* Park, 2000

*Halolaguna palinensis* Park, 2000: 241.

Distribution. China (Taiwan).

*Halolaguna sanmaru* Park, 2011

*Halolaguna sanmaru* Park, 2011: 201.

Distribution. Thailand.

*Halolaguna sublaxata* Gozmány, 1978

*Halolaguna sublaxata* Gozmány, 1978: 238.

Distribution. China (Hubei, Jiangsu, Liaoning, Shanxi, Taiwan, Zhejiang), Korea, Japan.

### Key to the Chinese *Halolaguna* species based on male genitalia

**Table:** 

1	Juxta with postero-lateral lobe about 1/2 length of juxta	**2**
–	Juxta with postero-lateral lobe as long as juxta or slightly longer than juxta	**4**
2	Aedeagus without cornutus	***Halolaguna guizhouensis***
–	Aedeagus with cornutus	**3**
3	Juxta nearly rounded; aedeagus with a rounded apex	***Halolaguna flabellata* sp. n.**
–	Juxta nearly square; aedeagus with a pointed apex	***Halolaguna oncopteryx***
4	Gnathos slender, longer than uncus	***Halolaguna sublaxata***
–	Gnathos obviously shorter than uncus	**5**
5	Aedeagus extending to a discal process distally	***Halolaguna discoidea* sp. n.**
–	Aedeagus not extending to a discal process distally	***Halolaguna palinensis***

#### Halolaguna discoidea sp. n.

Taxon classificationAnimaliaLepidopteraLecithoceridae

http://zoobank.org/E3FAFA75-8449-4793-AC0A-E7D624379185

**Figure 1.**
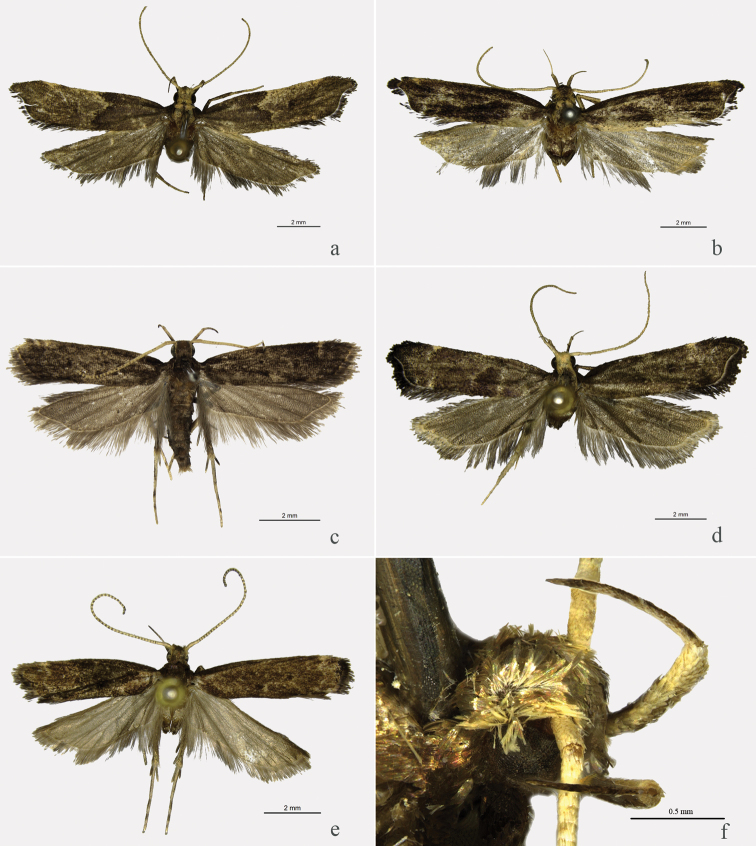
Male adults of *Halolaguna* species. **a** *Halolaguna discoidea* sp. n., paratype, Chongqing **b** *Halolaguna flabellata* sp. n., holotype, Guangxi **c** *Halolaguna guizhouensis*, Chongqing **d** *Halolaguna oncopteryx*, Chongqing **e** *Halolaguna sublaxata*, Zhejiang **f** *Halolaguna sublaxata*, Hubei, head from dorsolateral view.

**Figure 2.**
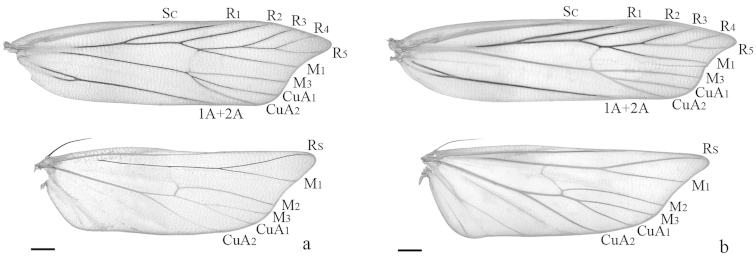
Wing venation of *Halolaguna* species. **a** *Halolaguna discoidea* sp. n., slide No. TKJ14008W **b** *Halolaguna flabellata* sp. n., slide No. ZYM06260W (Scales = 0.5 mm).

**Figure 3.**
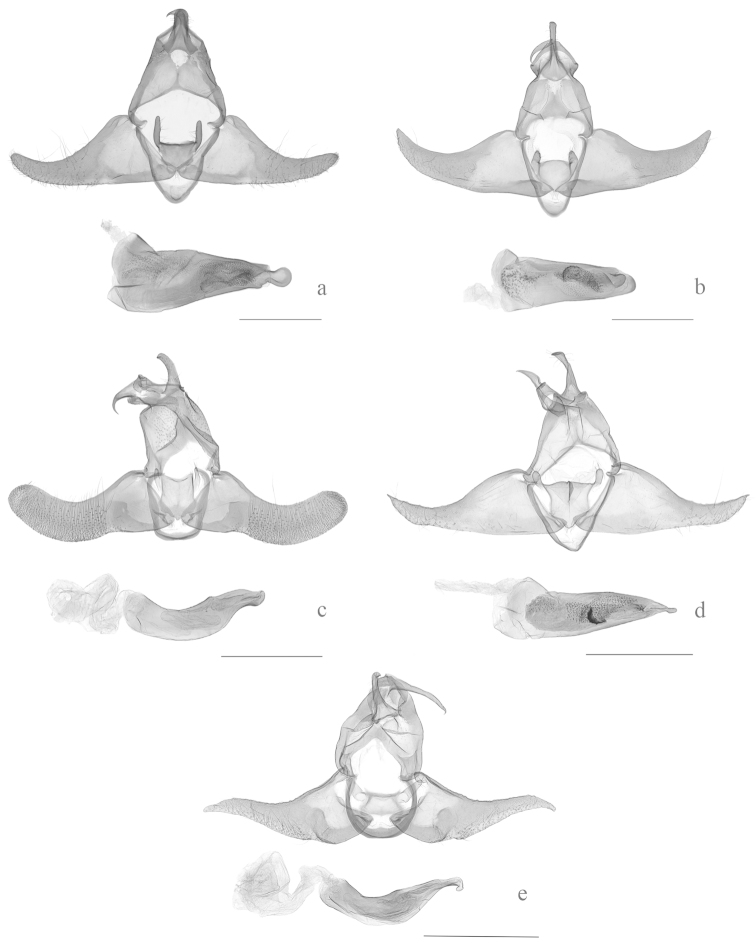
Male genitalia of *Halolaguna* species. **a** *Halolaguna discoidea* sp. n., slide No. WYQ13157 **b** *Halolaguna flabellata* sp. n., slide No. TKJ13034 **c** *Halolaguna guizhouensis*, slide No. TKJ13055 **d** *Halolaguna oncopteryx*, slide No. TKJ13039 **e** *Halolaguna sublaxata*, slide No. TKJ13051 (Scales = 0.5 mm).

**Figure 4.**
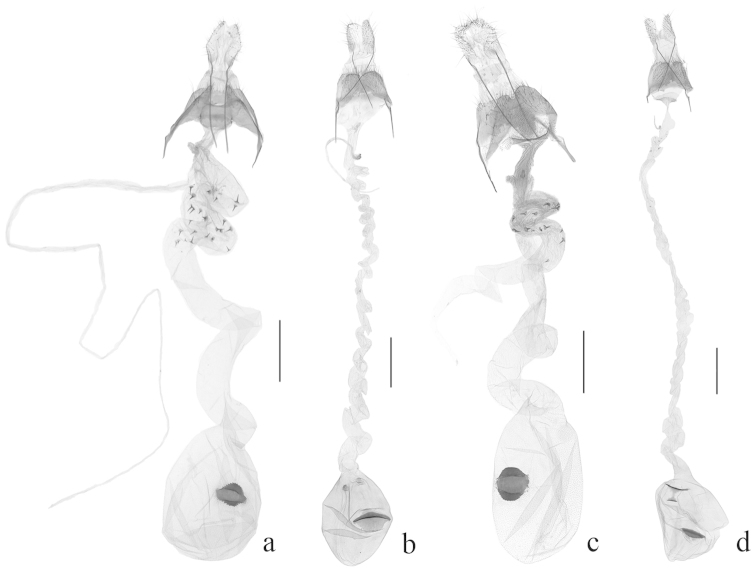
Female genitalia of *Halolaguna* species. **a** *Halolaguna discoidea* sp. n., slide No. TKJ14004 **b** *Halolaguna guizhouensis*, slide No. TKJ14087 **c** *Halolaguna oncopteryx*, slide No. TKJ13035 **d** *Halolaguna sublaxata*, slide No. TKJ14088 (Scales = 0.5 mm).

Figs 1a , 2a , 3a , 4a

##### Type material.

Holotype ♂, **China:** Tudiyan, Mt. Simian (28°60'N, 106°40'E), Chongqing, 1200 m, 15.vii.2012, leg. Yinghui Sun and Aihui Yin, genitalia slide No. TKJ13023. Paratypes: 1 ♂, Mt. Simian, Chongqing, 1000 m, 21.vii.2010, leg. Xicui Du and Shengwen Shi; 1 ♂, same locality, 22.vii.2010, leg. Xicui Du and Lifang Song, genitalia slide No. WYQ13157, venation slide No. TKJ14008W; 1 ♂, 2 ♀, Labahe (30°17'N, 102°29'E), Tianquan County, Sichuan Province, 1300 m, 28.vii.2004, 29.vii.2004, leg. Yingdang Ren; 1 ♀, Mt. Daming (23°24'N, 108°30'E), Nanning, Guangxi Zhuang Autonomous Region, 1200 m, 5.viii.2011, leg. Shulian Hao and Yinghui Sun, genitalia slide No. TKJ14004.

##### Diagnosis.

This species is similar to *Halolaguna oncopteryx* (Wu, 1994) and *Halolaguna flabellata* sp. n. in the forewing shape and the male genitalia, but can be separated from these by the juxta with thin claviform postero-lateral lobes slightly longer than the juxta, and the aedeagus with a discal process apically. In *Halolaguna oncopteryx* (Wu, 1994) and *Halolaguna flabellata* sp. n., the postero-lateral lobes of the juxta are short finger-shaped, about 1/2 length of the juxta, and the aedeagus is absent of discal process apically.

##### Description.

Adult (Figs 1a, 2a) with wing expanse 16.5–18.0 mm. Head yellowish white, with scattered brown scales. Antenna yellowish white, scape brown on ventral surface, flagellum with pale brown annulations. Labial palpus yellowish white, with scattered brown scales; second segment with appressed scales; third segment slender, about same length as second. Thorax brown, tegula purple brown. Forewing with costal margin almost straight from basal 1/4 to 3/4; apex protruding triangularly; termen oblique, concave below apex; ground color deep grayish brown; subapical spot yellowish white, nearly triangular; discal and discocellular spots blackish brown, nearly rounded; a yellowish white line extending from costal 2/5 to above fold, edged with blackish brown scales along inner margin, curved triangularly inward to outer margin of discal spot; cilia blackish brown, yellowish white basally; venation: R_3_ stalked with R_4+5_ for basal half of its length, R_4_ and R_5_ stalked for 2/3 length, R_5_ to termen, M_1_ and R_3+4+5_ from upper angle of cell, M_2_ absent, M_3_ from above lower angle of cell, CuA_1_ and CuA_2_ shortly stalked at base, from lower angle of cell, cell closed. Hindwing and cilia grayish brown, yellowish white basally; venation: Rs and M_1_ stalked for 2/5 length, M_3_ and CuA_1_ stalked for about 1/3 length, remote from M_2_, cell close partly. Fore leg with ventral surface brown, dorsal surface yellowish white, mottled brown scales, tarsus yellowish white on distal 1/3; mid leg yellowish white, mottled brown scales on ventral surface; hind leg blackish brown, yellowish white on dorsal surface of tibia and on distal half of tarsus.

Male genitalia (Fig. 3a): Uncus broad at base, narrowed to middle, distal half nearly parallel sided, bearing setae laterally, broadly rounded apically. Gnathos short, nearly triangular, curved distally, pointed apically. Valva broad at base, distinctly narrowed to middle, then slightly narrowed to narrowly rounded apex; costa gently concave beyond middle; ventral margin nearly straight. Sacculus narrow, reaching 1/3 length of dorsum. Juxta nearly quadrate, slightly convex antero-medially, almost straight posteriorly; postero-lateral lobe thin claviform, bearing setae laterally, bluntly rounded apically, longer than juxta. Vinculum narrow. Aedeagus stout, slightly longer than valva, broad basally, narrowed to apex; basal half with dense spinules, distal 2/5 with dense granules, apically produced to a discal process.

Female genitalia (Fig. 4a): Eighth sternite with caudal margin deeply concave in U shape at middle, bearing dense setae laterally. Posterior apophyses about twice length of anterior apophyses. Antrum inconspicuous. Ductus bursae long and heliciform, about four times length of corpus bursae, slightly narrow basally, with numerous thumbtack-shaped spinules ranging from basal 1/4 to 1/2; ductus seminalis slender and long, arising from basal 1/4 of ductus bursae. Corpus bursae oval; signum nearly oval, placed at middle of corpus bursae, margined with teeth anteriorly and posteriorly, medially concave, forming a broad and flat central groove.

##### Distribution.

China (Chongqing, Guangxi, Sichuan).

##### Etymology.

The name of this species is derived from the Latin adjective *discoideus* (discal), in reference to the discal process of the aedeagus at apex.

#### Halolaguna flabellata sp. n.

Taxon classificationAnimaliaLepidopteraLecithoceridae

http://zoobank.org/D3B12A57-853E-4B81-B865-F1A19028BAD1

Figs 1b , 2b , 3b

##### Type material.

Holotype ♂, **China:** Jinxiu County (24°07'N, 110°11'E), Guangxi Zhuang Autonomous Region, 650 m, 28.iv.2008, leg. Hui Zhen and Li Zhang, genitalia slide No. TKJ13034. Paratype: 1 ♂, Hongqilinchang (21°54'N, 107°54'E), Shangsi County, Guangxi Zhuang Autonomous Region, 260 m, 2.iv.2002, leg. Shulian Hao and Huaijun Xue, venation slide No. ZYM06260W.

##### Diagnosis.

This species is similar to *Halolaguna oncopteryx* (Wu, 1994) superficially and in the male genitalia, but can be separated from the latter by the valva with a blunt apex lacking an apical spine, the juxta nearly rounded, and the apex-rounded aedeagus with two sclerotized figs. In *Halolaguna oncopteryx*, the apex of the valva has a strong apical spine, the juxta is nearly square, and the apex-pointed aedeagus has one sclerotized fig.

##### Description.

Adult (Figs 1b, 2b): Wingspans 16.0–16.5 mm. Head brown, pale yellow on frons and around eye. Antenna yellowish white, with scattered pale brown scales. Labial palpus yellowish white, with scattered pale brown scales; second segment dark brown on outer surface, with appressed scales; third segment slender, slightly longer than second, pointed terminally. Thorax yellowish white, with brown scales medially; tegula purple brown. Forewing with costal margin almost straight from basal 1/5 to 4/5; apex protruding triangularly; termen oblique, slightly concave below apex; ground color dark brown; subapical spot pale yellow, nearly triangular; discal and discocellular spots blackish brown, small, nearly rounded (somewhat worn); cilia blackish brown, yellowish white basally; venation: R_3_ and R_4+5_ stalked for basal 1/3 length, R_4_ and R_5_ stalked for 3/5 length, R_5_ reaching termen, M_1_ and R_3+4+5_ shortly stalked at base, M_2_ absent, M_3_ and CuA_1+2_ from lower angle of cell, CuA_1_ and CuA_2_ shortly stalked, cell closed. Hindwing and cilia gray, yellowish white basally; venation: Rs and M_1_ stalked for basal 2/5 length, M_3_ and CuA_1_ shortly stalked, remote from M_2_ basally, cell close. Legs yellowish white; fore leg with femur having grayish brown scales on ventral surface, tibia purple brown, tarsus mottled dark brown scales; mid leg with scattered dark brown scales; hind leg dark brown on distal half of femur, at base of tibia and on basal half of tarsus.

Male genitalia (Fig. 3b): Uncus broadened in fan shape basally, clubbed distally, bearing short setae laterally, rounded apically. Gnathos narrow, basal 1/3 nearly aequilate, median portion gradually narrowed, distal 1/3 sharply narrowed to pointed apex. Valva broad at base, slightly narrowed to middle, distal half obviously narrowed, slightly curved dorsad distally, narrowly rounded apically; costa concave medially. Sacculus broad at base, narrowed distally, reaching 1/4 length of dorsum. Juxta nearly rounded, convex antero-medially, slightly arched posteriorly; postero-lateral lobe short thumb-shaped, bearing setae apically. Vinculum narrow. Aedeagus straight, shorter than valva, broad at base, slightly narrowed to rounded apex, with numerous unequally sized toothlike thorns at base, with dense spinules and granular teeth ranging from about middle to distal 1/4, distal half with two sclerotized irregular figs, one of them with teeth.

Female: Unknown.

##### Distribution.

China (Guangxi).

##### Etymology.

The specific name of this species is derived from the Latin adjective *flabellatus* (flabellate), in reference to the basally fan-shaped uncus.

#### Halolaguna guizhouensis

Taxon classificationAnimaliaLepidopteraLecithoceridae

Wu, 2012

Figs 1c , 3c , 4b

Halolaguna guizhouensis Wu, 2012: 394. Type locality: China (Guizhou).

##### Material examined.

**China:** Guizhou Province: 1 ♂, Linjiang (28°05'N, 105°32'E), Xishui County, 550 m, 26.ix.2000, leg. Haili Yu; Chongqing: 5 ♂, 1 ♀, Beipo (29°02'N, 107°11'E), Mt. Jinfo, 1100 m, 5.v.2013, 12.v.2013, leg. Xiaofei Yang; 1 ♂, same locality, 4.viii.2012, leg. Xiaofei Yang and Tengteng Liu; Guangxi Zhuang Autonomous Region: 2 ♂, 1 ♀, Shaopinglinchang (22°03’N, 106°55’E), Pingxiang, 280 m, 28.iii.2013, 2.iv.2013, 10.iv.2013, leg. Xiaofei Yang, genitalia slide No. TKJ14087♀; 1 ♂, Qinmu Village (24°59’N, 109°59’E), Yongfu County, 160 m, 1.v.2008, leg. Hui Zhen and Li Zhang; 1 ♂, Hekoubaohuzhan, Jinxiu County (24°07'N, 110°11'E), 650 m, 28.iv.2008, leg. Hui Zhen and Li Zhang, genitalia slide No. TKJ13055; 1 ♂, Xijiao (24°15'N, 108°01'E), Nandan County, Hechi, 868 m, 10.viii.2011, leg. Shulian Hao and Yinghui Sun; Guangdong Province: 1 ♂, Heshan (22°25'N, 112°32'E), 26.viii.2002, leg. Guilin Liu; 1 ♂, Hebao Island (21°52'N, 113°10'E), Zhuhai, 30 m, 18.v.2010, leg. Bingbing Hu and Jing Zhang.

##### Diagnosis.

Adult (Fig. 1c) with wing expanse 14.0–15.0 mm. This species is similar to *Halolaguna sublaxata* Gozmány, 1978 superficially by sharing small and rounded discal spot and relatively large fold and discocellular spots. It can be separated from the latter by the valva broadly rounded apically, the relatively short gnathos slightly shorter than the uncus, and the juxta with postero-lateral lobes shorter than the juxta in the male genitalia (Fig. 3c). In *Halolaguna sublaxata*, the valva is narrow and thin apically, the slender gnathos is distinctly longer than the uncus, and the postero-lateral lobes of the juxta are longer than the juxta.

Female genitalia (Fig. 4b): Eighth sternite bearing dense setae, with caudal margin slightly emarginated at middle. Anterior apophyses about 3/4 length of posterior apophyses. Ductus bursae about four times length of corpus bursae, long and heliciform; ductus seminalis slender, arising from basal 1/8 of ductus bursae. Corpus bursae nearly rounded; two small papillate signa placed posteriorly, with dense granules; one big rhombic signum placed at middle of corpus bursae, with a nearly triangular horizontal fig arising medially.

##### Distribution.

China (Chongqing, Guangdong, Guangxi, Guizhou).

##### Remarks.

*Halolaguna guizhouensis* was described by Wu (2012) based on two male specimens from Guizhou. The valva of this species is not distinctly narrowed distally, whereas the valva of its congeners is obviously narrowed to pointed apex. However, the venation of this species is consistent with that of the type species. The female is described here for the first time.

#### Halolaguna oncopteryx

Taxon classificationAnimaliaLepidopteraLecithoceridae

(Wu, 1994)

Figs 1d , 3d , 4c

Cynicostola oncopteryx Wu, 1994: 125. Type locality: China (Sichuan).
Halolaguna oncopteryx (Wu): Park 2000: 240.

##### Material examined.

**China:** Fujian Province: 1 ♂, 1 ♀, Mt. Meihua (25°20'N, 116°50'E), 19.vii.1988, 22.vii.1988, leg. Chinese Academy of Science; Chongqing: 1 ♂, 1 ♀, Mt. Simian (28°60'N, 106°40'E), 1000 m, 20.vii.2010, leg. Xicui Du and Lifang Song; Guangxi Zhuang Autonomous Region: 1 ♂, 1 ♀, Hongqilinchang (21°54'N, 107°54'E), Shangsi County, 260 m, 2.iv.2002, leg. Shulian Hao and Huaijun Xue; 2 ♂, 1 ♀, Shaoping linchang (22°03’N, 106°55’E), Pingxiang, 280 m, 19.iv.2012, 28.iii.2013, 13.iv.2013, leg. Xiaofei Yang; 2 ♀, Mt. Daming (23°24'N, 108°30'E), Nanning, 1200 m, 7.viii.2011, 8.viii.2011, leg. Shulian Hao and Yinghui Sun; Yunnan Province: 1 ♂, Tropical Botanical Garden (21°55'N, 101°17'E), Menglun County, 570 m, 13.viii.2005, leg. Yingdang Ren; Zhejiang Province: 1 ♀, Zhangkengkou (28°32'N, 118°99'E), Mt. Jiulong, 623 m, 5.vii.2013, leg. Aihui Yin and Xiuchun Wang; 2 ♂, 1 ♀, Neijiujian (28°40'N, 118°84'E), Mt. Jiulong, 430 m, 7.vii.2013, leg. Aihui Yin and Xiuchun Wang, genitalia slide No. TKJ13035♀; 1 ♂, 2 ♀, Yanping (28°38'N, 118°89'E), Mt. Jiulong, 530 m, 4.vii.2013, leg. Aihui Yin and Xiuchun Wang, genitalia slide No. TKJ13039♂; 2 ♂, 2 ♀, Huangtanyu (28°39'N, 118°84'E), Mt. Jiulong, 467 m, 8.vii.2013, leg. Aihui Yin and Xiuchun Wang; 1 ♂, Wuyanling (27°42'N, 119°39'E), Taishun County, 680 m, 28.vii.2005, leg. Yunli Xiao.

##### Diagnosis.

Adult (Fig. 1d) with wing expanse 15.0–16.0 mm. This species is similar to *Halolaguna sanmaru* Park, 2011 in the male genitalia, but can be separated from it by the valva with a strong apical spine, the juxta with postero-lateral lobes about 1/2 length of the juxta, and the aedeagus with a pointed apex (Fig. 3d). In *Halolaguna sanmaru*, the valva does not bear an apical spine, the postero-lateral lobes of the juxta are slightly longer than the juxta, and the aedeagus is rounded apically. This species is also similar to *Halolaguna discoidea* sp. n. in the female genitalia, but can be separated from it by the eighth sternite with caudal margin slightly concave at middle, and the ductus seminalis as broad as the ductus bursae (Fig. 4c). In *Halolaguna discoidea* sp. n., the caudal margin of the eighth sternite is deeply concave in U shape medially, and the ductus seminalis is slenderer than the ductus bursae.

##### Distribution.

China (Chongqing, Fujian, Guangxi, Sichuan, Taiwan, Yunnan, Zhejiang).

#### Halolaguna sublaxata

Taxon classificationAnimaliaLepidopteraLecithoceridae

Gozmány, 1978

Figs 1e , 1f , 3e , 4d

Halolaguna sublaxata Gozmány, 1978: 238. Type locality: China (Jiangsu).

##### Material examined.

**China:** Zhejiang Province: 1 ♂, Mt. Jiulong (28°29'N, 119°54'E), 400 m, 5.viii.2011, leg. Linlin Yang and Na Chen; 1 ♂, Houshanmen, Mt. Tianmu (30°15'N, 119°20'E), 500 m, 16.viii.1999, leg. Houhun Li et al.; Shanxi Province: 1 ♂, Mt. Li (35°26'N, 111°58'E), Jincheng, 1520 m, 16.viii.2006, leg. Xu Zhang and Haiyan Bai; Liaoning Province: 1 ♂, Shilizi (40°42’N, 124°42’E), Kuandian County, 10.viii.2009, leg. Weichun Li and Jiayu Liu; Hubei Province: 2 ♂, Mt. Wujia (31°05'N, 115°48'E), Yingshan County, 8.vii.2008, leg. Yunli Xiao, genitalia slide No. TKJ13051; 1 ♂, 2 ♀, Mt. Dahong (31°27'N, 113°00'E), Suizhou, 30.ix.2008, 1.x.2008, leg. Yunli Xiao, genitalia slide No. TKJ14088♀.

##### Diagnosis.

Adult (Fig. 1e, f) with wing expanse 14.0–15.0 mm. *Halolaguna sublaxata* Gozmány, 1978 can be separated from its congeners by the slender gnathos longer than uncus, and the valva slightly curved ventrad before apex in the male genitalia (Fig. 3e). *Halolaguna sublaxata* is similar to *Halolaguna guizhouensis* in the female genitalia by the corpus bursae sharing three signa, but can be separated from it by the position of the signa: in *Halolaguna sublaxata*, one large sub-triangular signum placed posteriorly, one small triangular signum below it, and the shuttle-shaped signum placed anteriorly (Fig. 4d); in *Halolaguna guizhouensis*, two small papillate signa placed posteriorly, and the third large rhombic signum is placed at middle of the corpus bursae.

##### Distribution.

China (Hubei, Jiangsu, Liaoning, Shanxi, Taiwan, Zhejiang).

## Supplementary Material

XML Treatment for Halolaguna discoidea

XML Treatment for Halolaguna flabellata

XML Treatment for Halolaguna guizhouensis

XML Treatment for Halolaguna oncopteryx

XML Treatment for Halolaguna sublaxata

## References

[B1] Gozmány L (1978) Lecithoceridae. In: Amsel HG Gregor F Reisser H (Eds) Microlepidoptera Palaeartica. Georg Fromme & Co., Wien, Volume 5, 238–239.

[B2] Li HH (2002) The Gelechiidae of China (I) (Lepidoptera: Gelechioidea). Nankai University Press, Tianjin, 504 pp.

[B3] van Nieukerken EJ Kaila L Kitching IJ Kristensen NP Lees DJ Minet J Mitter J Mutanen M Regier JC Simonsen TJ Wahlberg N Yen S-H Zahiri R Adamski D Baixeras J Bartsch D Bengtsson BÅ Brown JW Bucheli RS Davis DR De Prins J De Prins W Epstein ME Gentili-Poole P Gielis C Hättenschwiler P Hausmann A Holloway JP Kallies A Karsholt O Kawahara A Koster SJ CKozlov MV Lafontaine JD Lamas G Landry J-F Lee S Nuss M Park K-T Penz C Rota J Schmidt BC Schintlmeister A Sohn JC Solis MA Tarmann G Warren AD Weller S Yakovlev Y Zolotuhin VV Zwick A (2011) Order Lepidoptera Linnaeus, 1758. In: Zhang Z-Q (Ed.) Animal biodiversity: An outline of higher-level classification and survey of taxonomic richness.Zootaxa 3148: 212–221.

[B4] Park KT (2000) Lecithoceridae (Lepidoptera) of Taiwan (V): Subfamily Torodorinae: *Thubana* Walker, *Athymoris* Meyrick, *Halolaguna* Gozmány, and *Philharmonia* Meyrick. Insecta Koreana 17(4): 229–244.

[B5] Park KT (2011) A new species of *Halolaguna* Gozmány from Thailand (Lepidoptera: Lecithoceridae). Journal of Asia-Pacific Entomology 14: 201–203. doi: 10.1016/j.aspen.2010.12.006

[B6] Park KT Heppner JB Bae YS (2013) Two new species of Lecithoceridae (Lepidoptera, Gelechioidea), with a revised check list of the family in Taiwan. ZooKeys 263: 47–57. doi: 10.3897/zookeys.263.3781 23653516 10.3897/zookeys.263.3781 PMC3591763

[B7] Walker F (1864) List of the specimens of the lepidopterous insects in the collection of British Museum. Part XXIX & XXX, Tineites British Museum, London, 533–835, 837–1096.

[B8] Wu CS (1994) The Lecithoceridae (Lepidoptera) of China with descriptions of new taxa. Sinozoologia 11: 123–154.

[B9] Wu CS (1997) Fauna Sinica. Insecta, Lepidoptera: Lecithoceridae 7. Science Press, Beijing, 306 pp.

[B10] Wu CS (2000) A taxonomic study of the subfamily Torodorinae from Malaysia, with description of three new species (Lepidoptera: Lecithoceridae). Acta Zootaxonomica Sinica 25(4): 427–430.

[B11] Wu CS (2012) Lecithoceridae. In: Dai RH Li ZZ Jin DC (Eds) Insects from Kuankuoshui Landscape. Guizhou Science and Technology Publishing House, Guiyang, 392–396.

